# Using ChatGPT in Nursing: Scoping Review of Current Opinions

**DOI:** 10.2196/54297

**Published:** 2024-11-19

**Authors:** You Zhou, Si-Jia Li, Xing-Yi Tang, Yi-Chen He, Hao-Ming Ma, Ao-Qi Wang, Run-Yuan Pei, Mei-Hua Piao

**Affiliations:** 1 School of Nursing, Chinese Academy of Medical Sciences, Peking Union Medical College, No. 33 Badachu Road, Shijingshan District, Beijing, 100433, China, 86 13522112889

**Keywords:** ChatGPT, large language model, nursing, artificial intelligence, scoping review, generative AI, nursing education

## Abstract

**Background:**

Since the release of ChatGPT in November 2022, this emerging technology has garnered a lot of attention in various fields, and nursing is no exception. However, to date, no study has comprehensively summarized the status and opinions of using ChatGPT across different nursing fields.

**Objective:**

We aim to synthesize the status and opinions of using ChatGPT according to different nursing fields, as well as assess ChatGPT’s strengths, weaknesses, and the potential impacts it may cause.

**Methods:**

This scoping review was conducted following the framework of Arksey and O’Malley and guided by the PRISMA-ScR (Preferred Reporting Items for Systematic Reviews and Meta-Analyses extension for Scoping Reviews). A comprehensive literature research was conducted in 4 web-based databases (PubMed, Embase, Web of Science, and CINHAL) to identify studies reporting the opinions of using ChatGPT in nursing fields from 2022 to September 3, 2023. The references of the included studies were screened manually to further identify relevant studies. Two authors conducted studies screening, eligibility assessments, and data extraction independently.

**Results:**

A total of 30 studies were included. The United States (7 studies), Canada (5 studies), and China (4 studies) were countries with the most publications. In terms of fields of concern, studies mainly focused on “ChatGPT and nursing education” (20 studies), “ChatGPT and nursing practice” (10 studies), and “ChatGPT and nursing research, writing, and examination” (6 studies). Six studies addressed the use of ChatGPT in multiple nursing fields.

**Conclusions:**

As an emerging artificial intelligence technology, ChatGPT has great potential to revolutionize nursing education, nursing practice, and nursing research. However, researchers, institutions, and administrations still need to critically examine its accuracy, safety, and privacy, as well as academic misconduct and potential ethical issues that it may lead to before applying ChatGPT to practice.

## Introduction

Artificial intelligence (AI) was defined as a machine system that can make predictions, recommendations, and decisions influencing real or virtual environments based on a human-defined objective [1]. In recent years, with the rapid development of computer science, AI technology represented by machine learning, deep learning, and natural language processing has made amazing progress and achievements in the field of health care and been widely used in clinical practice, and has demonstrated a diagnostic performance that is not inferior to, or even better than human beings in some cases [2,3]. In the fields of nursing, AI is also playing an important role, including optimizing nursing processes [4], providing more personalized care [5], making health care more accessible [6], etc.

ChatGPT is an AI chatbot developed by OpenAI based on the third generation of the generative pretrained transformer architecture [7]. Since its release in November 2022, ChatGPT has attracted widespread attention and interest across the academic and scientific communities. Based on deep learning algorithms and natural language processing techniques, and trained with massive amounts of data from the internet, books, and articles, ChatGPT can automatically identify users’ inputs and generate appropriate responses to simulate the interactive dialogue and feedback process between humans [8]. In the field of clinical medicine, ChatGPT has exhibited its ability to assist in disease diagnosis, and it was reported the correct diagnosis rate of ChatGPT-3 was about 93.3% in 10 differential diagnoses [9]. At the same time, ChatGPT has also shown great potential in assisting nursing. For example, ChatGPT could help nurses to improve documentation by standardizing the terms and concepts, thus reducing nurses’ workload [10].

However, there are also widespread concerns about using ChatGPT in health care.

First, since ChatGPT’s training data came from the internet and lacked transparency, researchers have expressed concerns about its accuracy, usability, and safety in clinical practice [11]. Second, during clinical application, considering the potential inconsistency between the training data and the clinical application scenarios, ChatGPT may endure implicit bias and data-shift problems, as well as artificial hallucinations caused by them, which may lead to insecurity issues and care inequity [12,13]. Overreliance on ChatGPT can also weaken nurses’ judgment and lead to workforce deskilling. Third, in the academic publishing world, ChatGPT has caused broader discussions about academic integrity due to the difficulty of reviewers and available technologies in distinguishing content written by AI and a human [14]. In addition, especially in the field of education, although ChatGPT can help simplify administrative work, more educators expressed concerns that overdependence and complete trust in ChatGPT may cause and reinforce automation bias, and prevent students from developing abilities of critical thinking [15].

There have been extensive discussions about the application of ChatGPT in nursing. However, to date, no study has comprehensively summarized the perceptions on using ChatGPT in different nursing domains. Therefore, the aim of this study was to synthesize the opinions and acceptance of using ChatGPT from different application scenarios in nursing, as well as the strengths and weaknesses of ChatGPT and its possible impacts, to provide a reference for the future development of a large language model (LLM) that is more appropriate for nursing education and practice.

## Methods

### Study Design

This scoping review was conducted according to the 5-step methodological framework proposed by Arksey and O’Malley [16] (identifying the research question, identifying relevant studies, study selection, charting the data, and collating, summarizing, and reporting the results). The reporting of the review was guided by the PRISMA-ScR (Preferred Reporting Items for Systematic Reviews and Meta-Analyses extension for Scoping Reviews) guidelines [17].

### Identifying the Research Questions

1. How is ChatGPT used in different nursing fields, and what are the opinions and acceptance of this technology?
2. What are the strengths, weaknesses, ethical considerations, and potential impacts of the application of ChatGPT in nursing?

### Identifying Relevant Studies

A comprehensive literature search was conducted in 4 web-based databases (PubMed, Embase, Web of Science, and CINHAL) from 2022 to September 3, 2023, to identify studies reporting the opinions and acceptance of using ChatGPT in nursing fields. Two reviewers (YCH and XYT) screened the references of the included articles to further identify relevant studies.

To include as many studies as possible, the search terms were not limited strictly. The search terms in PubMed included two key topic areas: (“ChatGPT” OR “Chatbot*” OR “Large language model” OR “LLM” OR “LLMs”) AND (“Nursing” OR “Nurse*”). The search, using a combination of keywords and Boolean operators, was designed to comprehensively cover the intersection of ChatGPT and nursing.

### Study Selection

The inclusion criteria were as follows: (1) articles associated with the application or opinions of ChatGPT in nursing fields, such as nursing education, nursing practice, nursing academic writing, etc; (2) any types of articles including original articles, review articles, preprints, protocols, editorials, letters to editor, correspondence, and case reports; and (3) English publications. We excluded studies without available full-text and nonhuman studies.

All identified articles were first imported into the EndNote X9 (Clarivate Analytics) software to manually remove duplicates. Then, two reviewers (YZ and SJL) independently screened the titles and abstracts through the Rayyan application according to the inclusion and exclusion criteria to include studies for further full-text assessment. Any disagreements were resolved through consensus by consulting another reviewer (MHP).

### Charting the Data

According to the research question, two reviewers (XYT and YCH) independently extracted and synthesized pertinent information using an Excel sheet, including authors, year of publication, country, study design, objective of study, study results (opinions or findings of using ChatGPT in nursing), fields of concern, and suggestions or recommendations for future studies. Any disagreements were resolved through consulting another reviewer (MHP).

### Collating, Summarizing, and Reporting the Results

The PRISMA (Preferred Reporting Items for Systematic Reviews and Meta-Analyses) flow diagram showed the process of study selection. Two researchers (YZ and SJL) independently used an inductive approach to analyze and thematically summarize the contents of the included studies to identify the opinions and acceptance of similarities and differences about using ChatGPT in nursing. On this basis, the opinions extracted from studies were further synthesized and categorized according to different nursing fields in which ChatGPT was applied (such as nursing education, nursing practice, nursing research, nursing writing, etc). A table of supplement material in Multimedia Appendix 1 were also created to demonstrate the status and opinions of using ChatGPT in nursing.

## Results

### Search Results

**Figure 1.**
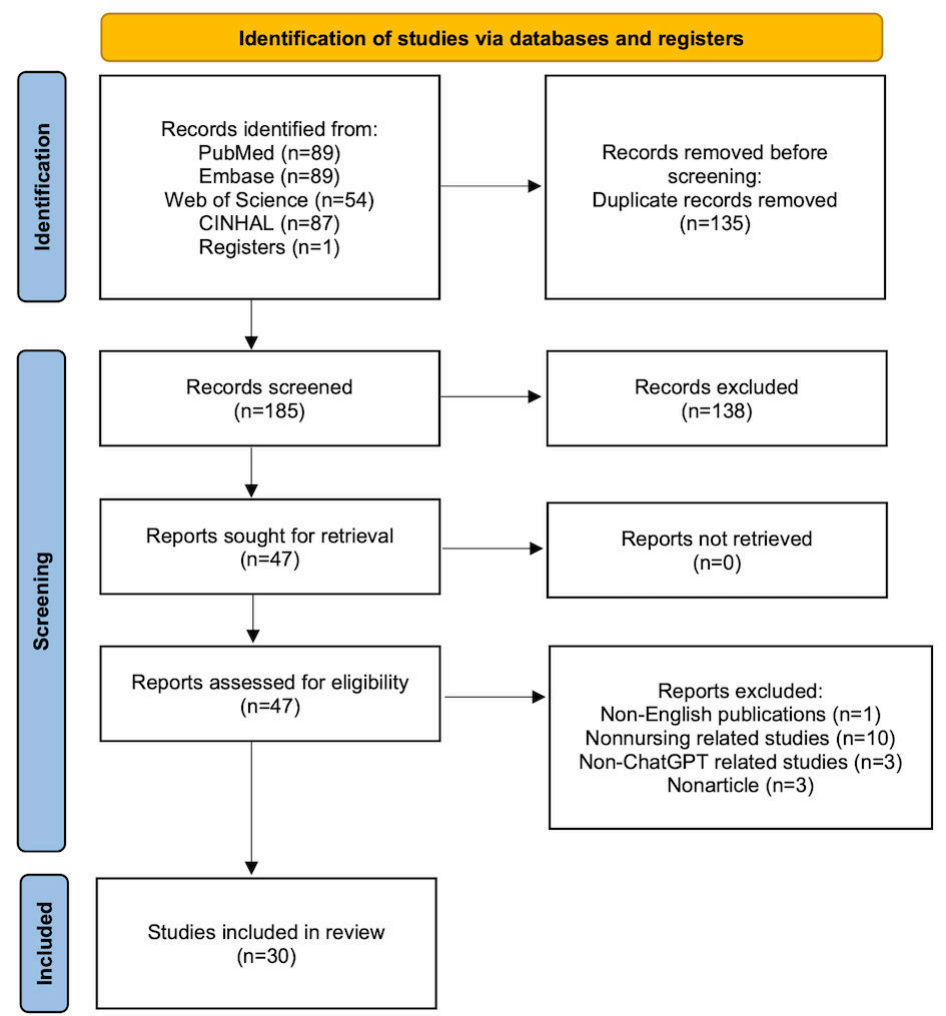
PRISMA flow diagram of study selection. PRISMA: Preferred Reporting Items for Systematic Reviews and Meta-Analyses.

Figure 1 showed the process of literature selection. A total of 320 studies were identified from the initial literature search. After removing the duplicates (n=135), 185 studies were identified for titles and abstracts screening, of which 47 studies meeting the inclusion criteria were allowed for full-text evaluation. Finally, 17 studies were excluded, and 30 studies were included in this review.

### Study Characteristics

Table 1 summarized the characteristics of the included studies. All 30 studies were published in 2023. The United States (7/30), Canada (5/30), and China (4/30) were countries with the most publications, accounting for more than 50% of all publications. In terms of study design, more than half of the studies were editorials (12/30) as well as letters to the editor (6/30), only 3 were original articles, and this study’s design was unclear in 7 studies. Table 2 presented the fields of concern of the included studies. Most studies focused on the application of ChatGPT in nursing education (n=20). Other fields of concern included using ChatGPT in nursing practice (n=10), nursing research (n=2), nursing academic writing (n=2), nursing examination (n=2), and nursing future (n=1). Six studies addressed the use of ChatGPT in multiple fields of nursing [15,18-22].

**Table 1.** Characteristics of included studies.

Characteristics		Studies, n (%)
**Year of publication**		
	2022	0 (0)
	2023	30 (100)
**Country**		
	United States	7 (23.33)
	Canada	5 (16.67)
	China	4 (13.33)
	Japan	2 (6.67)
	United Kingdom	2 (6.67)
	Australia	1 (3.33)
	Belgium	1 (3.33)
	Brazil	1 (3.33)
	Cambodia	1 (3.33)
	Indonesia	1 (3.33)
	Iraq	1 (3.33)
	Malta	1 (3.33)
	Netherlands	1 (3.33)
	Singapore	1 (3.33)
	Turkey	1 (3.33)
**Study design**		
	Editorial	12 (40)
	Not specific	7 (23.33)
	Letter to editor	6 (20)
	Article	3 (10)
	Debate essay	1 (3.33)
	Comment	1 (3.33)
**Main fields of concern**		
	Nursing education	20 (66.67)
	Nursing practice	10 (33.33)
	Nursing research	2 (6.67)
	Nursing academic writing	2 (6.67)
	Nursing examination	2 (6.67)
	Future nursing	1 (3.33)
	Multi-fields of nursing^a^	6 (20)

a Six studies addressed the use of ChatGPT in multiple fields of nursing [15,18-22].

**Table 2.** Fields of concern of the included studies.

Author	Year	Country	Study design	Nursing education	Nursing practice	Nursing research	Nursing academic writing	Nursing examination	Nursing future
Abdulai and Hung [18]	2023	Canada	Comment	✓	✓	✓	—^a^	—	—
Ahmed [23]	2023	Iraq	Letter	—	✓	—	—	—	—
Allen and Woodnutt [24]	2023	United Kingdom	Editorial	✓	—	—	—	—	—
Archibald and Clark [25]	2023	Canada	Editorial	✓	—	—	—	—	—
Berşe et al [19]	2023	Turkey	Letter	✓	✓	—	—	—	—
Castonguay et al [26]	2023	Canada	Not specific	✓	—	—	—	—	—
Chan et al [27]	2023	Hong Kong, China	Not specific	✓	—	—	—	—	—
Choi et al [28]	2023	Hong Kong, China	Not specific	✓	—	—	—	—	—
da Silva [29]	2023	Japan	Editorial	—	—	—	✓	—	—
Draganic [30]	2023	United States	Editorial	✓	—	—	—	—	—
Lim [31]	2023	United States	Editorial	✓	—	—	—	—	—
Frith [32]	2023	United States	Not specific	✓	—	—	—	—	—
Gunawan [33]	2023	Indonesia	Editorial	—	—	—	—	—	✓
Heerschap [20]	2023	Canada	Not specific	✓	✓	—	—	—	—
Irwin et al [21]	2023	Australia	Editorial	✓	✓	—	—	—	—
Kleebayoon and Wiwanikit [34]	2023	Cambodia	Letter	✓	—	—	—	—	—
Koo [35]	2023	Taiwan, China	Letter	✓	—	—	—	—	—
Moons and Van Bulck [22]	2023	Belgium	Editorial	—	✓	✓	—	—	—
O’Connor [36]	2023	United States	Editorial	✓	—	—	—	—	—
Odom-Forren [37]	2023	United States	Editorial	—	✓	—	—	—	—
Scerri and Morin [38]	2023	Malta	Editorial	—	✓	—	—	—	—
Shay [15]	2023	United States	Not specific	✓	✓	—	—	—	—
Siegerink et al [39]	2023	Netherlands	Editorial	—	—	—	✓	—	—
Sun and Hoelscher [40]	2023	United States	Article	✓	—	—	—	—	—
Taira et al [41]	2023	Japan	Article	—	—	—	—	✓	—
Tam et al [42]	2023	Singapore	Not specific	✓	—	—	—	—	—
Thakur et al [43]	2023	Canada	Letter	✓	—	—	—	—	—
Vitorino and Júnior [44]	2023	Brazil	Letter	✓	—	—	—	—	—
Woodnutt et al [45]	2023	United Kingdom	Debate essay	—	✓	—	—	—	—
Zong et al [46]	2023	China	Article	—	—	—	—	✓	—

a A blank space indicates that the content is not covered in the corresponding article.

### ChatGPT and Nursing Education

Existing research has shown that ChatGPT has great potential in the field of nursing education. For educators, ChatGPT can be used for curriculum development, drafting course materials, and generating practice tests, which can simplify teachers’ course preparation and assessment tasks [15,42,43]. Teachers can use ChatGPT to simulate patient encounters, providing students with an interactive learning experience to practice skills such as communication and assessment to enhance education [21,36,42]. For students, since ChatGPT has the function of instant feedback, it can be used as a tool to quickly acquire knowledge and skills, helping to improve learning efficiency and time management [19,27,40]. Students can also create individualized learning plans and obtain personalized feedback from ChatGPT, and use it to develop their writing skills, which will help motivate students to carry out independent learning and improve the efficiency and accuracy of the writing process [21,35,36,42-44]. In addition, ChatGPT has been believed to improve students’ digital literacy [26,42].

However, opposition exists at the same time. The researchers argue that using ChatGPT in nursing education may lead to plagiarism in assignments and academic dishonesty, given its superior ability to generate textual content [21,28,31,36]. It is also for this reason that, ChatGPT may undermine the nursing education assessment system that is now based on essays and assignments [24,36]. Students’ excessive use of ChatGPT may lead to reduced course participation [15]. Moreover, due to the nature of passive acceptance, over-reliance on ChatGPT will be detrimental to students’ ability to transform information into knowledge, as well as critical thinking, literature retrieval, and evidence synthesis [15,20,28,31,32,42].

### ChatGPT and Nursing Practice

The current view is that nurses can provide an unprecedented personalized care to patients based on ChatGPT; at the same time, patients can use it for health consultations, information about the status of their diseases and symptoms, and about their treatments [23]. In addition, due to the advantages of rapid assistance and rapid resource accessibility, ChatGPT can be used as a tool for nurses to quickly access information, helping nurses to keep up to date with information about patients’ illnesses, treatments, and medications, which is conducive to optimizing time management and providing high-quality care for patients [37,38,40].

However, despite the promising applications, there are still some problems and limitations in applying ChatGPT to nursing practice. First, ChatGPT cannot guarantee the security and confidentiality of the information uploaded to the servers. Therefore, inputting detailed and private information of patients to it may lead to a leakage of patients’ privacy [18-20,23,38]. Second, unlike search engines, ChatGPT does not search the internet to find the best answer to a question, but rather analyzes a large amount of data and then predicts the next most likely word in the answer, and therefore may output incorrect or biased information [19,20,37,38,45]. What’s more, nursing is a human-centered discipline, and a major disadvantage of chatbots is that they do not have the unique emotions and empathy of humans. Communication based on ChatGPT may make communication between nurses and patients impersonal and lacking in empathy, which may have a negative impact on the nurse-patient relationship [18,19,23,27,37,38].

### ChatGPT and Nursing Research, Writing, and Examination

There are also widespread concerns about using ChatGPT in academia and publishing. As ChatGPT is not an individual nor can it be held responsible for the content it generates, scholars argued that the decision to list ChatGPT as a coauthor was wrong and undesirable [39]. In addition, researchers had attempted to complete the nursing examinations using ChatGPT. Taira et al [41] found that ChatGPT demonstrated a stable, very close passing level in the 2019‐2023 Japanese National Nurse Examinations, however, ChatGPT showed some limitations in dealing with questions in complex situations. Zong et al [46] tested ChatGPT’s performance on the 2017‐2021 Chinese National Nurse Licensing Examination. The results showed that ChatGPT did not pass the examination in any of the years but scored equally close to the passing score [46].

## Discussion

### Principal Findings

This scoping review aimed to summarize the opinions and acceptance of published studies on the use of ChatGPT in nursing fields. The results of our study indicated that, nursing research on ChatGPT is still in its infancy and few original research has been conducted. ChatGPT has the potential to provide nursing students with personalized study guides, provide patients with high-level personalized care plans, and greatly facilitate research and academic writing efforts, but at the same time, it can also lead to automation bias, nurse-patient mistrust, and potential ethical issues caused by misinformation, and academic misconduct issues. Discussion about using ChatGPT in nursing education, nursing practice, and nursing research and academic writing remains heated and the researchers have not yet reached a unanimous opinion.

Considering the global nursing shortage, the cultivation of exceptional nurses has become an important issue in the field of nursing education. Therefore, when new technologies are available, what role they can play in nursing education is of particular interest. First, ChatGPT can assist teaching. For example, ChatGPT’s superior generative and analytical capabilities can help teachers reduce their workload by converting complex learning materials into easy-to-understand classroom content and assisting in grading students’ work [47]. Second, ChatGPT can facilitate changes in learning methods. ChatGPT can generate outlines to assist with literature reviews; create realistic clinical cases and scenarios to help medical students improve their diagnostic skills; and act as a personal tutor to create personalized learning plans and materials based on students’ abilities and learning feedback to improve learning efficiency [47,48]. In addition, ChatGPT was found to improve information skills in nursing students. In a study by Rahman and Watanobe [49], ChatGPT was found to assist students in generating code, checking code errors, and debugging and optimizing code. This is very important. With the advent of the digital age, programming will likely become a required course for nursing education and an essential skill for nurses in the future. ChatGPT’s significant help in programming learning is very meaningful to the learning of nursing informatics and cultivation of digital literacy for nursing students.

Although ChatGPT has demonstrated potential benefits in nursing education, opposition emerges from researchers. Academic writing is crucial for students’ success, yet crafting a research paper is a daunting task, even for experienced writers. ChatGPT plays a vital role in assisting with the writing process, but also raises issues about academic dishonesty, particularly when students become overly dependent on it [50]. In addition, students can also exploit ChatGPT for cheating during examinations, thus undermining the integrity of these assessments [51,52]. Furthermore, the use of ChatGPT in nursing education also brings ethical considerations such as data privacy and security. Students may share personal thoughts, feelings, and experiences while using ChatGPT, posing potential risks associated with the collection of this sensitive information [53].

Therefore, when integrating nursing education and the emerging technology, educators should comprehensively consider the strengths and limitations of ChatGPT. Educators and educational institutions should embrace this technology with an open mind and avoid simply banning its use. In practice, educators should teach students to critically evaluate and properly use ChatGPT to avoid overreliance; and use diverse teaching methods to encourage them to acquire skills of critical and independent thinking, and clinical reasoning. It is also critical to address and resolve ethical concerns, such as finding a balance between data privacy and correctly using ChatGPT. Moreover, educational institutions or educational administrations ought to establish guidelines and consensus or systems regarding the proper use of ChatGPT in nursing education.

In addition to nursing education, researchers also showed great interest in how ChatGPT can be applied to and improve nursing practice. ChatGPT empowers patients with health consultations and can help nurses to give personalized patient care by acting as an information tool. In a study by Kuroiwa et al [54], patients achieved accurate self-diagnosis of carpal tunnel syndrome and lumbar spinal stenosis by ChatGPT. ChatGPT seems to have the potential to become a patient self-management and condition monitoring tool outside the hospital. Therefore, future research could attempt to develop a ChatGPT-based chatbot and integrate it into existing mobile health (mHealth) intervention programs and platforms, exploring the role of mHealth interventions integrated with a LLM on symptom control and lifestyle change in patients with chronic diseases.

However, ethical concerns (ie, security and confidentiality, accuracy and bias in information output, and the lack of human empathy) also exist, and some issues are inevitable due to the nature of AI. For instance, the disclosure of patients’ privacy and provision of incorrect information may damage the trusting relationship between patients and nurses. Additionally, compassion emerges from interpersonal relationships and social interactions with persons, thus chatbots were considered to lack the capacity for compassion [55]. However, some consumer informatics studies found that chatbots seemed to be better at projecting the impression of empathy. In the study by Chen et al [56], a chatbot provided high-quality, empathetic, and easy-to-read answers to cancer-related questions on social media that were comparable to those provided by doctors. While the issue of empathy seems to be resolved, it is worth pondering whether chatbots will still be able to balance empathy and ethics to provide reliable answers to patients’ questions in the face of complex and varied real-life clinical environments and problems.

Given these concerns, implementing risk management strategies to control these risks is crucial. First, data confidentiality is essential when applying ChatGPT in nursing practice, and patients should be provided with informed consent and told not to disclose private personal information. Second, information provided by ChatGPT may be inaccurate and biased, thus professionals’ interventions such as reviewing the information developed by ChatGPT, and addressing bias in decision-making processes are necessary. Third, although ChatGPT can greatly improve nurses’ efficiency, it still cannot replace the important role of nurses. Future nurses should emphasize the human touch and ethical considerations in nursing processes and conduct more research to determine the support resources needed to effectively use this technology [19].

The concerns regarding using ChatGPT in other nursing fields also exist. As far as research and academic writing is concerned, several studies have now listed ChatGPT as a coauthor [36,57,58]. However, Palagani et al [59] found that although ChatGPT can generate article content as well as references as requested by the author, most of the references were incorrect or nonexistent. As a supportive tool for academic writing, ChatGPT can assist researchers in conducting a literature review and correcting grammatical errors to improve writing quality [60]. However, the abuse of ChatGPT may carry a great risk of leading to academic misconduct. In a study by Gao et al [14], reviewers indicated that it was difficult to distinguish between content generated by AI and human. Although recognition tools such as GPTZero and GPT-2 Output Detector (OpenAI) are already available, accurately identifying AI-generated content in submitted manuscripts will still be a daunting task as chatbot algorithms are iterated and optimized. Therefore, future research should focus on the development of recognition tools for AI-generated content and try to optimize the language style of different languages to improve the detection performance.

Scholars also explored ChatGPT’s capability to pass nursing licensing examinations and found that although it approached the passing threshold, it failed to meet the required passing standards. Considering that ChatGPT was developed primarily based on English-language data, and that there are differences in health care policies, regulations, languages, and cultures in various countries, this may partly explain why ChatGPT could not pass the examinations. This emphasizes an important ethical concern about the applicability and fairness of using AI in different health care settings. To address this issue, incorporating a wider range of languages and cultural contexts may be the future aim of AI technologies’ development.

### Future Directions

First, from the perspective of nursing education, educators should instruct students on the proper use of ChatGPT. Teachers should inform students to consciously consider LLMs such as ChatGPT as information search engines and learning assistants to avoid overreliance. Further, the most important thing is to cultivate students’ critical thinking and information discernment skills so that they can recognize artificial hallucination and extract useful information provided by ChatGPT while discarding untrue and false contents. Additionally, educational institutions could establish guidelines and consensus about the proper use of ChatGPT in nursing education to standardize the current state of using LLMs in the educational profession. Second, in the context of nursing practice, given the potential of applying ChatGPT into symptom management and lifestyle change in patients with chronic diseases, a ChatGPT-based chatbot could be developed and integrated into mHealth intervention programs, and patients’ private data can be secured by setting access rights and encrypting private data. In addition, more research and multiple efforts are required to identify the support resources needed to apply ChatGPT into nursing practice. Specifically, laws and regulations, and ethical standards for using LLMs in clinical practice are still to be introduced by the government and health care management agency; in terms of health care organizations, use guidelines and training curricula should be developed according to local application scenarios, patients’ needs, and nurses’ qualifications in the future; for researchers and developers, there is still a need for further diagnostic accuracy evaluation and usability testing to enhance the reliability of ChatGPT in complex clinical environments. Third, regarding nursing research, future research should concentrate on developing advanced tools to identify AI-generated content. To enhance the applicability and fairness of using ChatGPT, incorporating a broader spectrum of languages and cultural contexts may be the future aim of AI technologies’ advancement.

### Limitations

This study also had some limitations. First, this study only included publications in English, which may lead to a certain publication bias. Second, the search deadline for this study was September 3, 2023, considering the rapidly growing publication volume of studies on the application of ChatGPT in nursing, further reviews are still needed in the future to include more studies to enrich our findings. In addition, given the small number of original studies available about ChatGPT and nursing, this review included a wide range of types and quality of studies, and some of the low-quality studies may compromise the generalizability of the results of this study.

### Conclusions

As an emerging AI technology, ChatGPT has received a lot of attention and generated intense discussion in various nursing fields. Although at present, there is still a lack of original studies about its practical application in nursing, ChatGPT has showed great potential to revolutionize nursing education, nursing practice, and nursing research. However, before it can be applied to practice, researchers, institutions, and administrations still need to critically examine the privacy, safety, and accuracy as well as academic misconduct and potential ethical issues it may lead to.

## Supplementary material

10.2196/54297 Multimedia Appendix 1 Details on the study content of the included studies.

10.2196/54297 Checklist 1 PRISMA-ScR (Preferred Reporting Items for Systematic Reviews and Meta-Analyses extension for Scoping Reviews) checklist.

## Abbreviations

AI: artificial intelligence
LLM: large language model
mHealth: mobile health
PRISMA: Preferred Reporting Items for Systematic Reviews and Meta-Analyses
PRISMA-ScR: Preferred Reporting Items for Systematic Reviews and Meta-Analyses extension for Scoping Reviews
