# Supplementary material for: Using ChatGPT in Nursing: Scoping Review of Current Opinions
Source: JMIR Med Educ. 2024 Nov 19;10:e54297. doi: 10.2196/54297 (PMC11611787; doi:10.2196/54297)
Supplement: Multimedia Appendix 1 [file mededu-v10-e54297-s001.pdf]

Table S1. Details on the study content of the included studies.

| Author/Year/ Country | Study design | Objective | Opinions or findings | Fields of concern | Suggestions or recommendations |
|---|---|---|---|---|---|
| Abdulai et al., 2023, Canada[16] | Comment | The study reflected on how the use of ChatGPT may undermine the values, principles, and core assumptions that underpin nursing research, education, and practice. | ● ChatGPT cannot ensure the confidentiality of information, and may undermine the privacy and confidentiality inherent in nursing practice.<br>● ChatGPT may not capture human emotions, and priority the practical usefulness of computing over offering explanatory or theoretical analysis.<br>● ChatGPT may not be possible to identify or solve nursing problems that demand wisdom, critical thinking, intuition, and personal judgment.<br>● The reductionist approaches of ChatGPT may be overly simplistic and may not be capable of analyzing or understanding clients with complex health challenges or tailoring responses to individual cases.<br>● ChatGPT may facilitate academic dishonesty, undermines the moral value of integrity in nursing, and may produce nurses with less adherence to moral values, little or no sense of trustworthiness, and overconfidence in handling complex cases. | Nursing education<br>Nursing research<br>Nursing practice | ● It is important to critically reflect upon the potential and problematics of mixing AI data with a humanistic discipline like nursing.<br>● Until robust regulatory mechanisms on AI are developed, nursing must adopt some actionable strategies on ChatGPT that will ensure that the use of the AI tool does not distort the ethical values of nursing practice, education, and research. |

| | | | | | |
|---|---|---|---|---|---|
| Ahmed, 2023, Iraq[33] | Letter to editor | The study delved into the ramifications of ChatGPT within the nursing domain and accentuated its capacity and constraints to transform the discipline. | • Nurses can effectively employ ChatGPT to administer individualized care to patients on a level that was previously unattainable.<br>• ChatGPT has the potential to facilitate the development of chatbots, furnishing patients with pertinent information regarding their medical conditions and corresponding treatments.<br>• ChatGPT can serve as a tool to enhance the education and training of nurses, and have the potential to facilitate the creation of computer-based training modules that replicate authentic clinical situations.<br>• ChatGPT has the potential to serve as a resource for nurses to obtain immediate access to pertinent details regarding medications, therapies, and medical interventions.<br>• ChatGPT cannot substitute the personal interaction and empathetic care that nurses extend to their patients.<br>• ChatGPT may involve confidentiality and safeguarding issues. | Nursing practice | • It is crucial to contemplate the possible limitations and establish adequate measures to safeguard patient confidentiality and avert the technology from substituting human nurses. |

| | | | | | |
|---|---|---|---|---|---|
| Allen et al., 2023, UK[31] | Editorial | The study discussed the potential risk to assessments in nursing education due to the use of ChatGPT. | ● ChatGPT may potentially undermine the existing assessments in nursing education. | Nursing education | ● Educators should design curricula and assessment tasks for human intelligence, that better fit with the needs of clinical practice, and recognize the potential benefits that chatbots have both now and in the future. |

| | | | | | |
|---|---|---|---|---|---|
| Archibald et al., 2023, Canada[51] | Editorial | The study introduced and used ChatGPT, highlighted its likely impact on higher education, and what nursing and the health sciences can do about it. | ● The avoiding of ChatGPT may lead a lack of structures to ensure the integrity of student learning, make students lose the benefits of integrating ChatGPT into their learning, prevent them from analyzing shortcomings of ChatGPT critically. However, educators can avoid the risk of manifold and serious privacy and security concerns.<br>● Enforcing restrictive measures for ChatGPT will be resource intensive and ineffective. Students with personal ethics and academic integrity may respond with frustration to restrictive measures. Prohibition prevents educators from developing students' critical appraisal of ChatGPT's outputs, and may amplify the individual information seeking behaviors that occur in the absence of access to or support from nurses and other healthcare providers.<br>● The author aligned with and advocated to integrate ChatGPT into educational processes and assessments, and ChatGPT should be used following the principles of academic integrity. | Nursing education | ● It is likely viable to balance the privacy, security and academic integrity risks with the possible benefits of the application of ChatGPT.<br>● It is paramount to ensure a flexible process of assessment and resulting institutional approaches and policies to keeping pace with the current and rapidly evolving state of this AI technology. |

| | | | | | |
|---|---|---|---|---|---|
| Berşe et al., 2023, Turkey[17] | Letter to editor | The study aimed to increasing the awareness of nurse educators and nurses about the potential benefits, limitations, and risks of using ChatGPT. | ● ChatGPT can contribute to enhancing nurses' knowledge and skill levels, providing rapid and accurate information, and improving time management.<br>● ChatGPT have possible negative impacts on the nurse-patient relationship due to chatbots' inadequacy in emotional and empathetic communication.<br>● ChatGPT may provide inaccurate or biased information and cause issues regarding data privacy and security. | Nursing education<br>Nursing practice | ● More research is still needed in this area in the future, and it is recommended that future research focus on identifying the necessary training and support resources for nurses to effectively utilize this technology.<br>● Nurses should not overlook the significance of human touch and emotional connection while evaluating the advantages offered by technology. |
| Castonguay et al., 2023, Canada[28] | Not specific | The study discussed the opportunities, challenges, and future of using ChatGPT in nursing education. | ● ChatGPT has the potentials for students to facilitate learning, improve digital literacy, and encourage critical thinking about AI integration in healthcare.<br>● ChatGPT cannot replace the importance of fundamental human skills that are crucial to providing high-quality patient care. | Nursing education | ● Nurse educators can incorporate ChatGPT into their curriculum through formative or summative assessments and should prioritize faculty development to understand and use AI technologies effectively.<br>● Collaboration between educational institutions, regulatory bodies, and educators is crucial to establish provincial and national competencies and frameworks that reflect the increasing importance of AI in nursing education and practice. |

| | | | | | |
|---|---|---|---|---|---|
| | | | | | ● It is paramount that nurses and nurse educators be open to AI-enabled innovations as well as continue to critically think about their potential value to advance the profession, so nurses are better prepared to lead the digital future |
| Chan et al., 2023, HK, China[25] | Not specific | The study investigate students and teachers' reflections on ChatGPT by integrating it into nursing courses. | ● ChatGPT is helpful due to its quick response time and can assist with conducting interviews, however, it cannot provide empathetic and therapeutic communication.<br>● ChatGPT has the potential to enhance learning experience, however, there are concerns about information sources and confidentiality.<br>● The nurse educator support using ChatGPT as a search engine, however, the accuracy of the information generated by it still needs to be evaluated. | Nursing education | ● The issues of disclosure of patient or sensitive data must be considered when incorporating ChatGPT in teaching and student learning.<br>● Policies and guidelines for academic integrity should be established to prevent plagiarism.<br>● Educators should foster a learning environment that promotes students' critical reasoning skills. |
| Choi et al., 2023, HK, China[29] | Not specific | The study discussed the potential risks and benefits about the use of ChatGPT and gave nurse educators suggestions on how to teach student use it | ● Over-reliance on ChatGPT may prevent students from developing critical thinking and independent thought, and cause a misunderstanding of a concept or topic.<br>● ChatGPT might affect nursing students' communication and interactions with educators and peers. | Nursing education | ● Nurse educators should carefully review students' work to detect cheating, and there is a need to develop more sophisticated software to detect AI-generated written assignments.<br>● Nurse educators should view ChatGPT as tools to supplement traditional |

| | | | | | |
|---|---|---|---|---|---|
| | | critically and avoid over-reliance. | ● ChatGPT may lead to academic dishonesty and exacerbate equity problems.<br>● When critically used, ChatGPT might enhance learning process for nursing students, and facilitate continuing nurse education. | | teaching approaches, and a platform should be established to foster collaboration among nurse educators to share their experiences and best practices in addressing the use of ChatGPT in nurse education.<br>● Nursing educators should teach students to critically appraise and correctly use ChatGPT, and encourage them to acquire critical thinking skills, independent thought and clinical reasoning skills through diverse teaching methods to avoid over-reliance on ChatGPT.<br>● Nurse educators and students should know the limitations of AI technology and ensure that it is used in conjunction with other teaching methods to provide holistic nurse education. |
| da Silva, 2023, Japan[52] | Editorial | The study took a deeper look at the validity and ethics of authorship of a recently published article, in which authorship was shared with ChatGPT. | ● ChatGPT failed to provide any literature to support any of the statements in that text.<br>● ChatGPT did not satisfy any of the four ICMJE criteria for authorship.<br>● ChatGPT may distort publication record and bibliometric profile related to citations. | Nursing academic writing | ● It is important to deal with authorship-related policy now, before it is too late. |

| | | | | | |
|---|---|---|---|---|---|
| Draganic, 2023, USA[53] | Editorial | The study aimed to discuss the potential implications of ChatGPT for nurse practitioner education. | ● Certain aspects of the role of nurses, such as complex disease management, empathy, and compassion, cannot be replicated fully by chatbots.<br>● Nurses can use chatbots to complement and enhance the knowledge and skills, rather than being replaced and compromising the development of critical thinking skills. | Nursing education | ● Educators should embrace this new technology with both optimism and vigilance, exploring ways to integrate AI in a responsible manner to enhance our practice.<br>● Educators must remain cautious and ensure that technology did not compromise the development of critical thinking skills in healthcare, and technology should not replace human judgment and expertise. |
| Fidelindo, 2023, USA[30] | Editorial | The study offered the reflections on the impact of generative AI in nursing education. | ● Using ChatGPT does little to enhance recall of material and cannot transform information into knowledge for its passive nature.<br>● Using ChatGPT may give the illusion of understanding rather than actually learning.<br>● Students who rely on generative AI to answer reflective questions will be missing out.<br>● AI could cause plagiarism, undermining academic integrity. | Nursing education | ● Faculty can design active learning strategies beyond writing.<br>● Curriculum needs to balance academic and practical outcomes, use pedagogical technologies grounded in integrity and accountability.<br>● Schools must update their policies on academic integrity and amend plagiarism definitions to include generative AI.<br>● Publishing companies may require more stringent rules to discourage potential AI authors. |

| | | | | | |
|---|---|---|---|---|---|
| Frith, 2023, USA[32] | Not specific | The study reflected nurse educators' different perspectives about ChatGPT. | ● Nurse educators are concerned about the possibility of plagiarism and machine writing caused by ChatGPT, while some educators see it as a tool comparable to search engines, editing software, statistical software, and reference management systems.<br>● Students' abilities for conducting rigorous literature searches, synthesizing evidence, and attributing the work of others may be underdeveloped when ChatGPT is overused. | Nursing education | ● A multidisciplinary task force should be convened to provide guidance on meaningful use of AI.<br>● Faculty can encourage deep learning by teaching students to critically evaluate ChatGPT-generated information, make informed decisions about the accuracy of the information, and reflect on the use of the tool.<br>● Nurse educators should stay current on legislative actions to understand the policy and politics surrounding ChatGPT and other AI products. |

| | | | | | |
|---|---|---|---|---|---|
| Gunawan, 2023, Indonesia[54] | Editorial | The editorial aimed to provide a comprehensive overview of the future of nursing through asking ChatGPT. | ● The future of nursing in preventative care is likely to see a greater emphasis on “community-based care”, “technology integration”, and “evidence-based practice”.<br>● The future of nursing in technology integration is likely to see a greater emphasis on the use of digital tools and technologies.<br>● The future of AI in nursing is likely to involve the integration of AI-powered tools and technologies into various aspects of nursing practice.<br>● The future of robotics in nursing is likely to involve the integration of robotic assistants and telepresence robots into nursing practice.<br>● While robots and other forms of automation can help improve efficiency and reduce errors in care delivery, nurses will continue to play an important role in providing patient-centered care in the future.<br>● The future of nursing in interprofessional collaboration is likely to see a greater emphasis on teamwork, communication, and shared decision-making.<br>● The nurse image of the future is likely to be shaped by advances in technology, changes in | Future nursing | ● It is crucial for nurses to stay informed and adapt to new technologies and developments in the field of ChatGPT. |

| | | |
|---|---|---|
| | healthcare delivery models, and increasing diversity within the nursing profession.<br>• Male nurses are likely to involve an increased presence in the future of this profession. | |

| | | | | | |
|---|---|---|---|---|---|
| Heerschap, 2023, Canada[18] | Not specific | The study used ChatGPT and explored the use of AI and ChatGPT in wound care education and its potential benefits and current limitations. | ● Four possible use cases for LLMs in the field of wound care education including personalized education, accessible information, provision of up-to-date information, and multilingual support.<br>● LLMs may provide inaccurate or biased information that sounds correct.<br>● There is a concern regarding data privacy and inputting medical and patient data into the LLMs.<br>● The reliance for learners on AI may lead them not to take the time to develop critical thinking skills, and understand the foundational knowledge.<br>● The reliance on the use of chatbots in clinical decision-making may impact the patient trust and relationship with their healthcare provider.<br>● LLMs are as effective as the data in which they are trained upon, and this may lead to outdated and incorrect responses. | Nursing education<br>Nursing practice | ● Specialists within wound care must understand the impacts of LLMs and be prepared for its integration into clinical practice and education, while recognizing the current limitations and recognizing the need for further validation and testing of future tools as they are developed. |

| | | | | | |
|---|---|---|---|---|---|
| Irwin et al., 2023, Australia[19] | Editorial | The study discussed the implications of the age of AI for nursing and midwifery practice and education. | ● ChatGPT can simplify teaching work and processes, deliver authentic assessments and track students' performances, assist to build simulation scenarios quickly and affordably.<br>● Students can receive personalized feedback and remote support from ChatGPT, which can improve their writing motivation and personal learning skills and use NLP models to develop their writing skills.<br>● Academic integrity, specifically plagiarism is most likely the biggest challenge facing education institutions in relation to ChatGPT.<br>● Students or novice nurses could compromise patient safety if ChatGPT was inappropriately utilized within clinical practice without knowledge of the limitations.<br>● A lack of consensus on the use and application of NLP models within higher education may serve to widen the distance between industry and education as well as further social inequities. | Nursing practice<br>Nursing education | ● It is time to consider the expansion of the curriculum, incorporate data analytics and machine learning into undergraduate learning and be considered business as usual. |

| | | | | | |
|---|---|---|---|---|---|
| Kleebayoon et al., 2023, Cambodia [55] | Letter to editor | The study commented on the potential value of employing AI in nursing education. | ● The potential value of employing AI in nursing education was overemphasized.<br>● It might be unethical to use ChatGPT to create, assess and accept primary material without user engagement.<br>● ChatGPT needs a fresh design in order to work properly.<br>● Instead of simply banning the use of ChatGPT, educating students on the importance of academic integrity and teaching them how to use chatbot technology ethically in their learning process will be a preferable approach. | Nursing education | ● ChatGPT code of conduct needs to be reviewed improved and updated before being used in practice, study or instruction. |
| Koo, 2023, Taiwan, China[26] | Letter to editor | The study illustrate the potential of ChatGPT in nursing education and how to harness it. | ● The ability of chatbots to give real-time feedback may motivate students to engage in self-directed learning.<br>● Students should always verify chatbot-generated information with other reliable sources of information.<br>● Chatbots can be viewed as a tool to enhance the efficiency and accuracy of the writing process, and used as a supplementary tool to provide step-by-step procedures for problems involving calculations. | Nursing education | ● It is highly essential to develop critical thinking skills and emphasize ethical and responsible use of chatbots during nursing education.<br>● Guidelines for the proper use of chatbots as a learning tool need to be established in conjunction with traditional instruction. |

| | | | | | |
|---|---|---|---|---|---|
| Moons et al., 2023, Belgium[20] | Editorial | The study aims to inform readers about this technology, explore the potential use of ChatGPT for cardiovascular clinic and research and highlight potential misuse and errors. | ● ChatGPT has the potential to revolutionize the way we practice and conduct research in cardiovascular nursing, and its ability to generate natural language text, summarize large amounts of data, and answer specific questions makes it a valuable tool for healthcare professionals.<br>● The misuse of ChatGPT have cause concerns about academic plagiarism and deception both in educational institutions and academic world and publishing industry.<br>● The capacity of the AI system is regularly exceeded. | Nursing practice<br>Nursing research | ● It is important for healthcare professionals to stay up-to-date with the latest tools and techniques, including AI.<br>● It needs to be tested how and to what extent AI could improve patient care, and trials could be conducted to evaluate if evidence-based practice is better implemented if nurses are supported by an AI language model such as ChatGPT instead of only relying on typical scientific databases. |
| O'Connor et al., 2023, USA[23] | Editorial | The study used ChatGPT in academic writing and discussed how could ChatGPT be used in nursing education. | ● AI chatbots have the potential to enhance education by providing personalized, interactive learning experiences for students.<br>● AI chatbots can help prevent and avoid plagiarism, but also can cause plagiarism if not used responsibly and ethically.<br>● The use of AI software could lead to an erosion of the quality of the assessment, with implications for the development of the nursing profession. | Nursing education | ● The more diverse types of assessment used in nursing education and more sophisticated plagiarism software are needed to reduce and identify plagiarism.<br>● The risks of using AI tools should also be made clear to nursing students.<br>● It is important to educate nursing students about academic integrity and the value of gaining knowledge and skills from focused in-depth reading, critical thinking, and scientific writing. |

| | | | | | |
|---|---|---|---|---|---|
| Odom-Forren, 2023, USA[34] | Editorial | The study used ChatGPT and discussed the role of ChatGPT in perianesthesia nursing. | ● ChatGPT can provide instant, accurate, and personalized responses to a wide range of health care questions, whicn can save valuable time.<br>● ChatGPT can help nurses stay up-to-date on the latest research and best practices in perianesthesia nursing.<br>● While ChatGPT can help nurses provide better care, it cannot replace the human touch and compassion that are so essential to nursing.<br>● ChatGPT and other AI language models do have limitations and sometimes give incorrect answers.<br>● ChatGPT is not yet ready to be used a source of reliable information. | Nursing practice | ● Nurses must continue to provide holistic, patient-centered care and use ChatGPT as a supplement to their clinical knowledge and expertise.<br>● As we continue to explore the possibilities of AI in health care, it is important to embrace these new technologies and use them to enhance, rather than replace, the vital work of perianesthesia nurses.<br>● In terms of publication, the important take-home is that tools used for creation of information should be used appropriately and transparently. |

| | | | | | |
|---|---|---|---|---|---|
| Scerri, 2023, Malta[35] | Editorial | The study used ChatGPT and discussed the potential use, the benefits and the limitations and the implications of using ChatGPT in nursing practice. | ● ChatGPT could reduce repetitive writing and administrative work, provide case summaries or care plans, enhance communication, generate instructions and recommendations, translate medical language into easier to understand text and patients' native language, and simplify complex instructions.<br>● The overreliance on ChatGPT can lead to deskilling nurses, make interactions more impersonal and less therapeutic.<br>● Responses given by ChatGPT may not be reliable or evidence based, which could increase the risk of giving inaccurate or biased information to patients or other staff.<br>● ChatGPT is not able to ensure the confidentiality of the information being shared with it, and the information processed by ChatGPT is stored temporarily on OpenAI's servers and is not guaranteed to be secure.<br>● Chatbots like ChatGPT are currently limited and require further development to be effectively implemented in supporting the planning and delivery of nursing practice. | Nursing practice | ● Nurses need to take appropriate measures to protect any sensitive or confidential data related to the patient or the healthcare organization.<br>● While ChatGPT may improve nurses' efficiency, nurses must recognize they are just a tool, and cannot replace the role of nurses to provide the patient a 'human touch' and a 'therapeutic environment', and safe and effective care. |

| | | | | | |
|---|---|---|---|---|---|
| Shay, 2023, USA[12] | Not specific | The study aimed to summarize the potential applications and limitations of ChatGPT for faculty, students, and patients. | ● AI based tools may help simplify routine faculty administrative, course preparation, and assessment tasks.<br>● ChatGPT can provide self-tutoring and produce low-level multiple-choice practice tests with answers for students.<br>● Students' over-reliance on ChatGPT may decrease their active engagement with courses and prevent development of skills necessary to personally appraise, analyze, and synthesize data.<br>● ChatGPT may write plausible-sounding but incorrect answers to health queries by the public. | Nursing education<br>Nursing practice | ● Faculty and students should conduct open communication about the potential use and misuse of ChatGPT and ongoing dialog about its impact on teaching, learning, and patient health.<br>● It is important for nurses to remain informed about AI and help lead policy efforts to ensure the risks and benefits of AI serve the interests of patients and the public. |
| Siegerink et al., 2023, Netherlands[37] | Editorial | The study aimed to illustrate that it is wrong to refer to ChatGPT as an author of academic papers and highlights the concepts of accountability and contributorship. | ● LLMs, irrespective of their level of complexity, cannot be listed as an author or contributor for it cannot held accountable for the content of the work and is not an individual.<br>● As texts are structured and protocolized, LLMs can be used without much issue and the use of it is not needed mentioned; as texts where the arguments themselves are the core of the written work, the contribution of LLMs should be mentioned.<br>The decision to list "ChatGPT" as an author, was erroneous. | Nursing academic writing | ● A critical attitude and transparency are key in uses of LLMs in science and nursing education. |

| | | | | | |
|---|---|---|---|---|---|
| Sun et al., 2023, USA[24] | Article | The study aimed to illustrate the advantages and limitations of ChatGPT and raise recommendations for faculty to use it ccorrectly in nursing education. | ● Advantages of ChatGPT: rapid assistance, rapid resource accessibility, writing assistance, research support, examination preparation/study material generation, examination question response/rationale, proofreading and editing support, summarization assistance, clinical documentation generation.<br>● limitations of ChatGPT: accuracy, citations and references, limited up-to-date information, contextual understanding, medical terminology/jargon, exam question generation, bias, lack of personal reflection, non-text information analysis, word limit, acceptance as a source. | Nursing education | ● Faculty should understand the potential benefits and limitations of ChatGPT and create assignments that emphasize self-reflection, critical thinking, problem solving, and independent learning. Students must be taught how to critically evaluate information and how to make informed decisions. |
| Taira et al., 2023, Japan[38] | Article | The study aimed to evaluate the performance of ChatGPT on the Japanese National Nurse Examinations. | ● The 5-year average percentage of correct answers for ChatGPT was 75.1% for basic knowledge questions and 64.5% for general questions.<br>● The highest percentage of correct answers on the 2019 examination was 80% for basic knowledge questions and 71.2% for general questions.<br>● ChatGPT had a lower percentage of correct answers in some areas, such as pharmacology, social welfare, related law and regulations, endocrinology, metabolism, and dermatology, and a higher percentage of correct answers in the | Nursing examination | ● In the future, if additional data in the areas of poor performance are acquired and tuned, it is highly likely that the passing criteria will be exceeded in a stable manner. |

| | | |
|---|---|---|
| | areas of nutrition, pathology, hematology, ophthalmology, otolaryngology, dentistry and dental surgery, and nursing integration and practice. | |

| | | | | | |
|---|---|---|---|---|---|
| Tam et al., 2023, Singapore [21] | Not specific | This article discussed the challenges and implications of AI powered chatbot in nursing education. | ● Educators can ask ChatGPT to draft course materials and administrative paperwork.<br>● Students can leverage ChatGPT's searching and summarizing features for personalized self-paced learning experience.<br>● AI-Chatbot can be used to simulate patient encounters and provide students with opportunities to practice communication, assessment, and intervention skills.<br>● Over-reliance on ChatGPT may cause issues with plagiarism and limit critical thinking skills.<br>● AI-Chatbot technology could transform the nursing profession by aiding and streamlining administrative tasks.<br>● ChatGPT could be used to socially assist patients and for therapeutic purposes in mental health.<br>● AI-Chatbots can help nursing students and researchers to overcome technical barriers in nursing informatics. | Nursing education | ● Educators must proactively adjust their curricula and pedagogy, and provide clear guidelines on appropriate use and emphasize the importance of critical thinking and proper citation.<br>● With ChatGPT's potential for nursing research and education, faculty should consider how to use the time saved and engage nursing students to think critically and make decisions that benefit patients. |

| | | | | | |
|---|---|---|---|---|---|
| Thakur et al., 2023, Canada[22] | Letter to editor | This article discussed role of ChatGPT for curriculum development in nursing education. | ● AI is rapidly advancing in healthcare and nursing education.<br>● ChatGPT is an innovative AI platform which can facilitate curriculum development.<br>● Interactive features can aid educators in developing personalized education plans. | Nursing education | ● Future research can focus on its role in curriculum design, pedagogy, and simulation-based learning. |
| Vitorino, 2023, Brazil[27] | Letter to editor | The study used ChatGPT and aimed to discusse how will the ChatGPT impact contemporary nursing teaching? | ● The positive impacts of the ChatGPT on contemporary nursing education included enhancing student learning, improving clinical decisionmaking, facilitating collaboration and communication, supporting personalised learning, and enhancing accessibility and flexibility.<br>● The negative impact of using ChatGPT on contemporary nursing education included dependence on technology, information overload, limited human interaction, privacy and security concerns, bias, and accuracy.<br>● The potential challenges of using ChatGTP in contemporary nursing care education included technical proficiency, information management, curriculum development, student engagement and ethical considerations. | Nursing education | ● Nurse professors will need to be proactive in addressing these challenges to ensure that AI is integrated into nursing education to enhance student learning and support the development of critical thinking skills and clinical decision-making abilities.<br>● With the inevitable advancement of AI, we will have to learn how to work with it and not be against it. |

| | | | | | |
|---|---|---|---|---|---|
| Woodnutt et al., 2023, UK[36] | Debate essay | The study aimed to use ChatGPT to create a mental health nursing care plan and evaluate the quality of the output against the authors' clinical experience and existing guidance. | ● ChatGPT was able to provide a care plan that incorporated some principles of dialectical behaviour therapy, but the output had significant errors and limitations and thus there is a reasonable likelihood of harm if used in this way.<br>● There is a lack of policy and research to safeguard people receiving care - and this needs to be in place before AI should be used in writing nursing care.<br>● AI use may diminish mental health nurses' ability to provide safe care in its current form.<br>● Many aspects of mental health recovery are linked to relationships and social engagement, however AI is not able to provide this. | Nursing practice | ● Use of AI in writing nursing care plans should be avoided until a point where policy and guidance can safeguard the wellbeing of care recipients and the sophistication of AI output has increased.<br>● Further research evaluating AI output is needed to consider how AI may be used safely in care delivery. |
| Zong et al., 2023, China[39] | Article | This study aimed to evaluate the performance of ChatGPT on National Medical Licensing Examination, National Pharmacist Licensing Examination, and National Nurse Licensing Examination (NNLE) in China. | ● ChatGPT failed to pass the threshold score (60%) in NNLE from 2017 to 2021. The highest score was 0.5897 in 2017 and the lowest was 0.4612 in 2020 in NNLE. | Nursing examination | ● ChatGPT may require additional training data in non-English languages to enhance its performance in non-English medical exams.<br>LLMs need public benchmarking datasets and fair evaluation metrics for performance assessment, and need to interact with human experts from multiple dimensions and obtain continuous feedback. |

| | |
|---|---|
| | • The use of LLMs must also consider data privacy, cognitive bias, and comply with regulations. |

**Abbreviations:** AI, artificial intelligence; ChatGPT, Chat Generative Pre-trained Transformer; LLMs, large language models.
